# Proteomics analysis of periplaque and chronic inactive multiple sclerosis lesions

**DOI:** 10.3389/fnmol.2024.1448215

**Published:** 2024-08-21

**Authors:** Jordan M. Wilkins, Kiran K. Mangalaparthi, Brian C. Netzel, William A. Sherman, Yong Guo, Alicja Kalinowska-Lyszczarz, Akhilesh Pandey, Claudia F. Lucchinetti

**Affiliations:** ^1^Department of Neurology, Mayo Clinic, Rochester, MN, United States; ^2^Department of Laboratory Medicine and Pathology, Mayo Clinic, Rochester, MN, United States; ^3^Department of Bioinformatics and Computational Biology, University of Minnesota, Minneapolis, MN, United States; ^4^Department of Quantitative Health Sciences, Mayo Clinic, Rochester, MN, United States; ^5^Department of Neurology, Division of Neurochemistry and Neuropathology, Poznan University of Medical Sciences, Poznan, Poland; ^6^Department of Neurology, The University of Texas at Austin, Austin, TX, United States

**Keywords:** differentially expressed proteins, multiple sclerosis, pathology, protein networks, proteomics, spatial profiling

## Abstract

**Background:**

Multiple sclerosis (MS) is a demyelinating disease of the central nervous system characterized by increased inflammation and immune responses, oxidative injury, mitochondrial dysfunction, and iron dyshomeostasis leading to demyelination and axonal damage. In MS, incomplete remyelination results in chronically demyelinated axons and degeneration coinciding with disability. This suggests a failure in the ability to remyelinate in MS, however, the precise underlying mechanisms remain unclear. We aimed to identify proteins whose expression was altered in chronic inactive white matter lesions and periplaque white matter in MS tissue to reveal potential pathophysiological mechanisms.

**Methods:**

Laser capture microdissection coupled to proteomics was used to interrogate spatially altered changes in formalin-fixed paraffin-embedded brain tissue from three chronic MS individuals and three controls with no apparent neurological complications. Histopathological maps guided the capture of inactive lesions, periplaque white matter, and cortex from chronic MS individuals along with corresponding white matter and cortex from control tissue. Label free quantitation by liquid chromatography tandem mass spectrometry was used to discover differentially expressed proteins between the various brain regions.

**Results:**

In addition to confirming loss of several myelin-associated proteins known to be affected in MS, proteomics analysis of chronic inactive MS lesions revealed alterations in myelin assembly, metabolism, and cytoskeletal organization. The top altered proteins in MS inactive lesions compared to control white matter consisted of PPP1R14A, ERMN, SIRT2, CARNS1, and MBLAC2.

**Conclusion:**

Our findings highlight proteome changes in chronic inactive MS white matter lesions and periplaque white matter, which may be crucial for proper myelinogenesis, bioenergetics, focal adhesions, and cellular function. This study highlights the importance and feasibility of spatial approaches such as laser capture microdissection-based proteomics analysis of pathologically distinct regions of MS brain tissue. Identification of spatially resolved changes in the proteome of MS brain tissue should aid in the understanding of pathophysiological mechanisms and the development of novel therapies.

## 1 Introduction

A hallmark of multiple sclerosis (MS) is the presence of inflammatory demyelinating lesions disseminated in the central nervous system (CNS). Gradual irreversible damage over time results in injury to the CNS making MS one of the most common causes of non-traumatic disability in young adults. The disease is heterogeneous with several factors involving the immune system, genetics, and environment contributing towards progression. Profound heterogeneity and classification of four immunopathological patterns in early active MS lesions suggests there are various mechanisms of demyelination (Lucchinetti et al., 2000). Active lesions are considered correlates of disease relapses. However, not every active lesion will result in a relapse. Eventually, inactive plaques are predominantly observed in the chronic phase of MS (Frischer et al., 2015). In chronic MS, it has been suggested that pathogenic pathways converge representing a more consistent route of demyelination (Breij et al., 2008), which may signify underlying molecular mechanisms driving disease progression. Inactive white matter plaques present with a sharply demarcated border with few to no active microglia or macrophages (Frischer et al., 2015). While remyelination in the MS brain is well described, the extent of recovery is highly variable, incomplete, and is often followed by an additional round of demyelination (Prineas et al., 1993; Patani et al., 2007; Bramow et al., 2010). Thus, identifying molecular changes within chronic inactive white matter lesions and the surrounding area may provide valuable insight into key pathophysiological mechanisms.

To date, reports of proteomics analysis of brain tissue derived from MS patients are limited (Elkjaer et al., 2022). While omics-based approaches provide useful insight, the importance of interrogating tissue based on anatomical features is becoming increasingly recognized. For instance, proteomics analysis of microdissected MS lesions from acute plaques, chronic active plaques, and chronic plaques identified coagulation factors implicated in inflammation and MS pathology (Han et al., 2008). Similarly, proteome analysis of microdissected MS brain tissue from regions of chronic demyelination, remyelination, and periplaque white matter (PPWM) suggested changes in the extracellular matrix, oxidative stress, and the myelin sheath (Ly et al., 2011). Microdissected MS brain tissue from acute active and chronic active lesions identified EphrinB3, which is thought to inhibit oligodendrocyte progenitor cells affecting remyelination (Syed et al., 2016). Implementation of MALDI imaging of MS lesions led to the identification of thymosin beta-4 largely expressed at the rim of plaques where it may be involved in restorative processes (Maccarrone et al., 2017). Taken together, these studies demonstrate that different histopathologically defined regions in MS brain tissue have unique proteome signatures emphasizing the importance of studying molecular changes that are spatially distinct. Here, we utilized laser capture microdissection (LCM) and proteomics to identify molecular changes that occur in chronic inactive lesions and PPWM of MS patients compared to the white matter of control individuals with no apparent neurological disorders. Comparative analyses of proteins altered in various regions of MS brain tissue versus corresponding samples from controls further exemplifies the need to interrogate spatial changes to gain deeper insight into distinct pathophysiological mechanisms.

## 2 Materials and methods

### 2.1 Brain tissue

This study was approved by the Mayo Clinic Rochester Institutional Review Board. We included three CNS autopsy cases diagnosed with chronic MS (two females and one male) and three normal controls (three males) with no known neurological disorders (Supplementary Table 1). Histopathological examination of formalin-fixed paraffin-embedded (FFPE) MS tissue confirmed regions of interest corresponding to inactive demyelination, PPWM, and non-demyelinated cortex. Proteolipid protein 1 (PLP1) was used to visualize myelin and confirm no myelin debris products within the lesions. Macrophages were detected using CD68 staining.

### 2.2 Laser capture microdissection and sample processing of brain tissue for quantitative proteomic analysis

Tissue sectioning and mounting was performed at the Mayo Clinic Pathology Research Core. All FFPE tissue blocks were sectioned at 10 μm thick, placed onto polyethylene-naphthalate (PEN)-membrane slides, and dried. Slides were deparaffinized using standard xylene and ethanol rinses. To facilitate cutting, tissue was stained with a 0.5% (w/v) cresyl violet stain in 75% ethanol solution. Excesses stain was removed using a series of ethanol washes and allowed to dry. Microdissection was carried out on a ZEISS PALM MicroBeam laser microscope (Oberkochen, Germany). Regions of interest were captured in lysis buffer (100 mM Tris HCl, pH 8.0, 0.005% Z3-16) and frozen on dry ice followed by storage at −80°C. In our study, a single autopsy section (10 μm thick) was sufficient for proteomics analysis. On average after protein extraction, we achieved 0.29 μg/mm^2^ for control cortex, 0.27 μg/mm^2^ for control white matter, 0.25 μg/mm^2^ for MS cortex, 0.24 μg/mm^2^ for MS periplaque white matter, and 0.17 μg/mm^2^ for MS inactive lesions requiring approximately 6.8 mm^2^ to 12.1 mm^2^ of FFPE tissue for proteomics analysis. All the samples were adjusted to 2% SDS and crosslinks were reversed by heating at 98°C for 45 min with shaking. Following this, sonication was performed for 15 cycles with 30 seconds on and 30 seconds off using BioRuptor Pico (Diagenode). The samples were reduced with 5 mM TCEP and heated to 98°C for 45 min followed by sonication. Subsequently, the samples were alkylated with 10 mM iodoacetamide for 30 min in the dark. Tryptic digestion was performed using a single pot SP3-based workflow (Hughes et al., 2019). Briefly, samples were mixed with the SP3 beads to a final concentration of 1 μg/μL and incubated on a thermomixer at 1,800 rpm for 20 min. Beads were then washed with 70% ethanol followed by acetonitrile on a magnetic rack. The beads were resuspended in 50 mM TEABC and subjected to overnight on-bead trypsin digestion at 37°C on a thermomixer. The beads were then pelleted by centrifugation and supernatant was collected using a magnetic rack. The samples were further washed with water incubated for 5 min for complete recovery of peptides and pooled with the supernatant after digestion. Peptides were dried down and stored at −80°C until LC-MS/MS analysis.

### 2.3 Liquid chromatography with tandem mass spectrometry analysis and database searching

Orbitrap Exploris 480 mass spectrometer connected online to a Vanquish Neo liquid chromatography system was used for LC-MS/MS analysis (ThermoFisher Scientific, Bremen, Germany). Peptides were reconstituted in 0.1% formic acid (solvent A) and loaded onto a trap column (Optimize Technologies). Peptides were further separated on an analytical column (EasySpray 15 cm × 75 μm, C_18_ 2 μm, 100 Å; Thermo Scientific, San Jose, CA) with solvent B (acetonitrile, 0.1% formic acid) using a gradient of 3% to 25% for 115 min and to 35% for 10 min. The overall run time of peptide separation was 150 min. After each injection, 4 zebra wash cycles were performed followed by equilibration of both trap and analytical columns. While the peptides were being eluted from the analytical column, the mass spectrometer was operated in a data dependent mode with cycles of MS1 followed by MS2 every 2 seconds. In the MS1 scan, precursor ions were surveyed in the Orbitrap analyzer for a scan range of 350–1400 m/z with 120,000 resolution, 50 ms injection time and 300% normalized AGC target. For MS2 analysis, precursor selection range was limited to 350–1200 m/z and the most abundant precursor ions were sequentially isolated by quadrupole mass filter using 1.2 m/z isolation width. The precursor ions were subsequently fragmented using HCD collision mode with 30% normalized collision energy. The fragment ions were analyzed in the Orbitrap analyzer with 30,000 resolution, 100 ms injection time and 100% normalized AGC target. Monoisotopic precursor selection, charge state filter of 2–6 and intensity threshold of 30,000 were enabled for precursor selection for MS2. Dynamic exclusion of 30 seconds was enabled to prevent repeated MS2 fragmentation of the precursor ions. Internal calibration was performed using polysiloxane ion 445. 1203 m/z. Raw data was analyzed using Andromeda search engine in MaxQuant software suite (version 1.6.17) (Cox and Mann, 2008). Database searching was performed against the human protein database using trypsin cleavage specificity and two missed cleavages. The first search peptide tolerance of 20 ppm and main search peptide tolerance of 4.5 ppm was specified. Dynamic modifications used include oxidation at methionine, protein N-terminus acetylation and static modifications included carbamidomethylation at cysteine. The label free quantification algorithm was used for the protein abundance. Matches between the runs was enabled with an alignment time window of 20 min and a match time window of 0.7 min. Finally, identifications were filtered at 1% peptide-level and protein-level FDR.

### 2.4 Data analysis

Statistical analysis of the preprocessed proteomics data was carried out using the R package DEP (Differential Enrichment analysis of Proteomics data version 1.18.0) (Zhang et al., 2018). In brief, the data was log_2_-transformed and filtered so that a protein was identified in all replicates of at least one group. The data was then normalized using variance stabilized transformation. The remaining missing values were imputed using the “MinProb” function within DEP prior to differential analysis. The MinProb function imputes missing values with random draws from a left-shifted distribution of observed values.

### 2.5 Enrichment analysis and plots

For pathway and functional analysis, differentially expressed proteins with a *p*-value < 0.01 and a fold change ≥ 2 were considered. Significantly altered proteins were uploaded to the STRING database (Search Tool for the Retrieval of Interacting Genes/Proteins version 11.5) and biological processes (Gene Ontology, GO) and KEGG pathways (Kyoto Encyclopedia of Genes and Genomes, KEGG) were used (Szklarczyk et al., 2021). Ingenuity Pathway Analysis (IPA, QIAGEN Inc^1^.) was used for the generation of focused networks (Krämer et al., 2013).

The Venn diagram was generated using the R package VennDiagram (version 1.7.3). The clustered heatmap was generated in DEP (see data analysis section above). For the clustered heatmap, only the top altered proteins were considered (adjusted *p*-value < 0.05 and fold change ≥ 2). Keyword enrichment for the cluster heatmap was performed using DAVID (Database for Annotation, Visualization and Integrated Discovery; release December 2021) (Sherman et al., 2022).

The circular heatmap was generated using OriginPro 2024 (OriginLab Corporation, Northampton, MA, USA). The dendrogram on the circular heatmap was generated using the group average cluster method and Euclidean distances. The protein-protein network was generated using the STRING app within Cytoscape (version 3.10.2) (Shannon et al., 2003). The top 100 interacting proteins (filtered by degree level determined within Cytoscape) were used for the protein-protein network. Node size is scaled based on the degree level.

### 2.6 Upstream analysis

Three tools were utilized for the prediction of upstream regulators. Differentially expressed proteins in inactive white matter lesions compared to white matter controls (*p*-value < 0.01 and fold change ≥ 2) were utilized for the analysis. Kinases and transcription factors were predicted using KEA3 (Kinase Enrichment Analysis 3) and ChEA3 (ChIP-X Enrichment Analysis 3), respectively (Keenan et al., 2019; Kuleshov et al., 2021). For KEA3 and ChEA3, proteins were sorted by the mean rank score, which is a composite score calculated based on the enrichment of kinases and transcription factors identified across all libraries used in the analysis (Keenan et al., 2019; Kuleshov et al., 2021). For molecules in which the activation status was predicted, we used IPA upstream regulator analysis to sort molecules by their predicted activation Z-score (*p*-value < 0.01) (Krämer et al., 2013).

## 3 Results

### 3.1 Description of tissue

**FIGURE 1:**
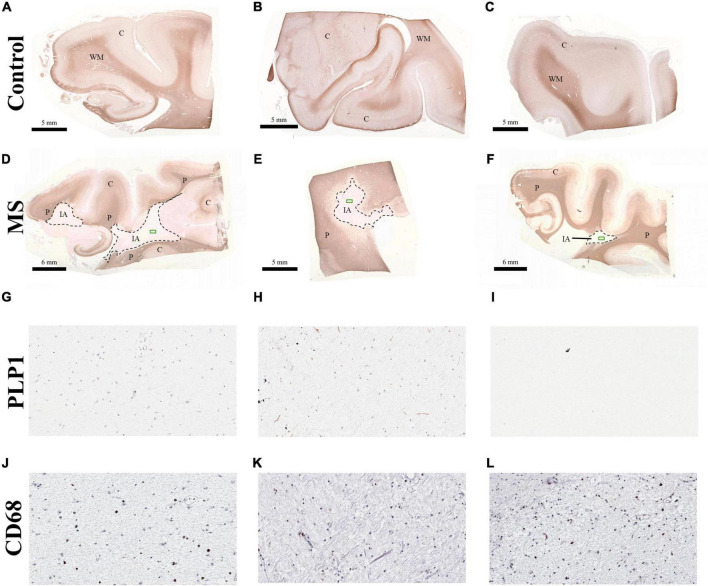
Immunohistochemical myelin staining of brain tissue used in study. All sections were stained for myelin proteolipid protein 1 (PLP1) and for macrophages using CD68. Tissues from control individuals are seen in **(A–C)** and multiple sclerosis patients in **(D–F)**. The green box indicated in **(D-F)** indicates the zoomed in regions of PLP1 **(G–I)** and CD68 **(J–L)** stains. Minimal cortex was present in MS case **E** and omitted from analysis. Brain tissue regions are indicated as follows: C, cortex; IA, inactive lesions; P, periplaque white matter; WM, white matter.

Formalin-fixed paraffin-embedded brain tissue from three chronic MS cases (median age and disease duration = 60 years and 22 years, respectively) and three control individuals (median age = 50) was used in this study (Figure 1, Supplementary Table 1). All control individuals had no apparent neurological disorders. Corresponding immunohistochemical myelin staining (myelin proteolipid protein, PLP1) was used to evaluate demyelination. A total of 14 regions were dissected from FFPE brain tissue (six from controls and eight from MS). White matter and cortex were present in all control tissues (Figures 1A–C). The MS brain tissue presented with inactive plaques and PPWM (Figures 1D–F). Minimal cortex was identified for one of the MS samples (Figure 1E), thus, providing only two cortex samples for analysis. Inactive plaques and PPWM in MS tissue was defined as previously described (Popescu et al., 2017). A close up of the PLP1 (Figures 1G–I) and CD68 (Figures 1J–L) staining confirms the lack of myelin debris and low presence of macrophages indicating no active demyelination in the lesion.

### 3.2 Spatial proteome alterations in multiple sclerosis brain tissue

**FIGURE 2:**
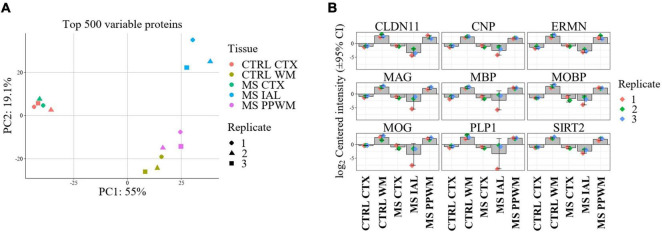
Proteome alterations are unique in MS lesions and periplaque white matter. Overview of proteome changes in multiple sclerosis brain tissue. **(A)** Principal component analysis of the top 500 variable proteins. **(B)** Bar plots showing significantly altered myelin-associated proteins in MS lesions compared to control and MS brain tissue regions. The data is centered around the log_2_-intensity. CTRL, control; CTX, cortex; IAL, inactive lesion; MS, multiple sclerosis; PPWM, periplaque white matter.

Label free quantitative proteomics analysis of laser capture microdissected brain tissue subregions led to the identification of 3,351 proteins. After filtering so that a protein was identified in all replicates of at least one group, a total of 3,099 proteins remained. Missing values were imputed prior to differential analysis (see Methods section). Principal component analysis (PCA) of the top 500 variable proteins shows good separation and clustering of chronic inactive lesions, PPWM, and control white matter (Figure 2A). The cortex of all samples clustered tightly but were distinct from white matter tissue (Figure 2A). As a quality control measure, we first assessed changes in proteins involved in myelin biosynthesis of the CNS previously implicated in MS (Figure 2B; Steinman, 1996; Kaushansky et al., 2010; Rice et al., 2012; Nave and Werner, 2014; Salek Esfahani et al., 2019; Raasakka and Kursula, 2020; Shen et al., 2021). As expected, all myelin-associated proteins show an enrichment in the control white matter compared to control cortex. More so, all chronic inactive MS lesions had significantly reduced myelin-associated proteins when compared to control white matter (*p*-value < 0.01 and a fold change ≥ 2, Figure 2B). These results support spatially distinct alterations in the proteome of MS lesions and PPWM compared to control white matter.

### 3.3 Chronic inactive white matter lesion proteome alterations

**FIGURE 3:**
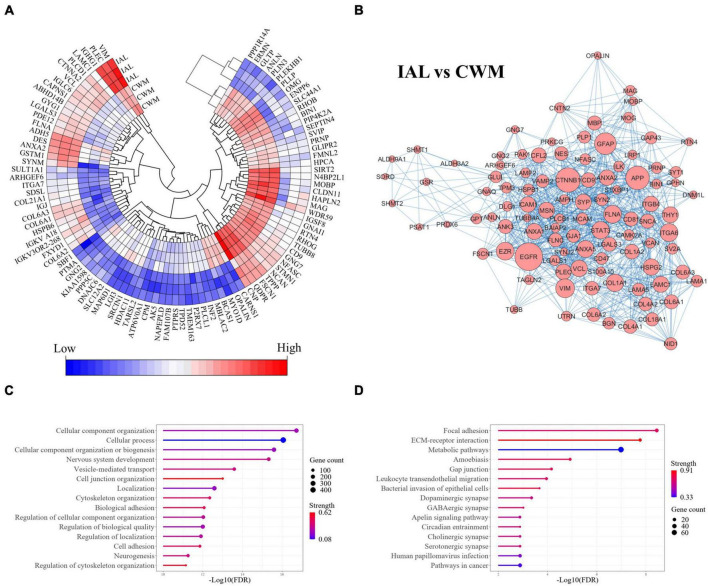
Cellular organization, biogenesis, and metabolism are altered in MS lesions. Proteome changes in MS inactive white matter lesions compared to control white matter. All proteins were significantly altered in MS white matter lesions when compared to control white matter tissue (*p*-value < 0.01 and fold-change ≥ 2). **(A)** A heatmap highlighting the top 100 differentially expressed proteins in MS inactive lesions when compared to control white matter tissue (ranked by *p*-value). All differentially expressed proteins can be seen in Supplementary Figure 1. **(B)** Protein-protein interaction network of differentially expressed proteins. The top 100 interconnected proteins are shown for clarity. A full interaction network can be seen in Supplementary Figure 2. **(C)** Enrichment of biological functions. **(D)** Enrichment of pathways. Gene count and Strength were determined by the STRING application in **(C,D)**. CWM, control white matter; IAL, inactive lesion; MS, multiple sclerosis.

The comparison of chronic inactive lesions to control white matter identified 504 significantly altered proteins (*p*-value < 0.01 and a fold change ≥ 2). Of the 504 altered proteins, 218 were upregulated and 286 were downregulated in the inactive lesions. The top differentially expressed proteins are shown in Figure 3A (see Supplementary Figure 1 for all changes). The STRING platform, which is a database of protein-protein interactions, was used to infer altered biological functions and pathways (Szklarczyk et al., 2021). An interaction network of the top interconnected proteins altered in MS lesions when compared to control white matter is shown in Figure 3B emphasizing the complex nature of changes occurring (see Supplementary Figure 2 for full network). Evaluation of the significantly altered proteins in MS lesions suggests that the top biological processes (Gene Ontology, GO) altered include cellular component organization and/or biogenesis, cellular process, and nervous system development (Figure 3C). The top pathways (KEGG) altered in inactive lesions compared to control white matter consisted of focal adhesion, ECM-receptor interaction, and metabolic pathways (Figure 3D). These findings highlight complex proteome changes occurring in MS lesions affecting cellular organization, signaling, development, and metabolism.

### 3.4 Periplaque white matter proteome alterations

Identifying alterations occurring in PPWM compared to inactive lesions and control white matter may help provide insight into early pathological mechanisms leading to plaque formation. Analysis of differentially expressed proteins in PPWM when compared to inactive lesions revealed similar changes as detected in the comparison of MS lesions to control white matter (Supplementary Figure 3). A total of 302 differentially expressed proteins were detected with 193 upregulated and 109 downregulated in PPWM when compared to inactive lesions (Supplementary Figure 3A). A protein-protein interaction network showed good interconnectivity amongst the 302 differentially expressed proteins (Supplementary Figure 3B). The top associated biological processes altered included regulation of cellular component biogenesis and organization, cellular process, and nervous system development (Supplementary Figure 3C). Perturbed pathways included bacterial invasion of epithelial cells, ECM-receptor interaction, and metabolic pathways (Supplementary Figure 3D).

**FIGURE 4:**
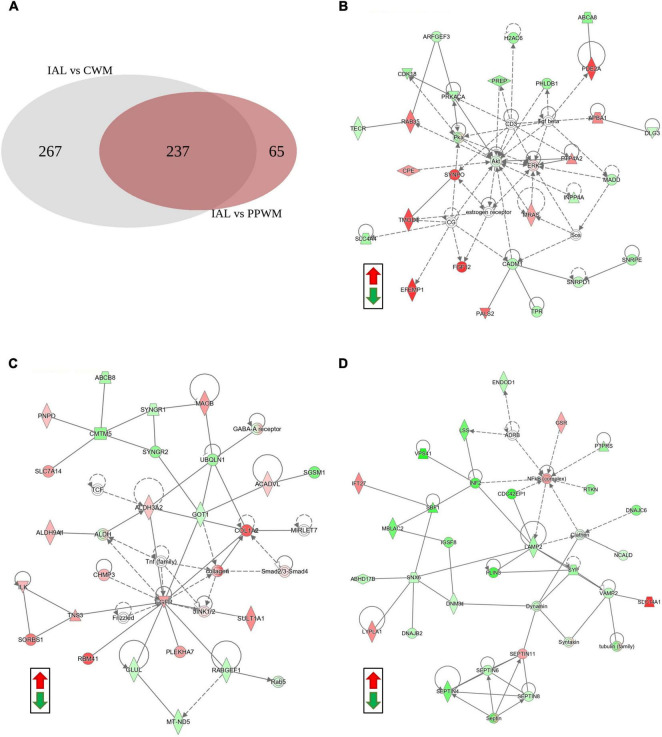
Network analysis reveals potentially unique alterations in periplaque white matter. Possible proteome changes that occur early in the white matter of MS brain tissue. **(A)** A Venn diagram showing the distribution of differentially expressed proteins amongst two comparisons in the white matter tissue (Inactive lesion versus control white matter, left; Inactive lesion versus periplaque white matter, right). **(B)** The top network perturbed within the 65 unique proteins likely affected in periplaque white matter. **(C)** The top network altered among the 267 unique proteins affected in chronic inactive lesions. **(D)** The top network altered in the 237 commonly affected proteins within MS inactive lesions. Green and red depict significantly decreased or increased proteins, respectively. Differentially expressed proteins were considered when the *p*-value < 0.01 and fold change ≥ 2. Abbreviations: CWM, control white matter; IAL, inactive lesion; PPWM, periplaque white matter.

To further help identify differentially expressed proteins that may be more specific to PPWM alterations in MS brain tissue, we examined the overlap of the two differential analyses (inactive lesions versus control white matter, and inactive lesions versus periplaque white matter) (Figure 4A). This comparison highlighted 65 proteins that may be uniquely altered in PPWM as compared to inactive lesions and control white matter (Figure 4A, right). Network analysis of these 65 unique PPWM alterations suggests that the top diseases and functions altered include developmental disorder, hereditary disorder, and neurological disease (Figure 4B). Similarly, 267 proteins were unique to inactive lesions when compared to control white matter (Figure 4A, left). The top network altered suggests changes in amino acid metabolism, cell morphology, and small molecule biochemistry (Figure 4C). Likewise, 237 proteins were commonly altered within inactive lesions with respect to PPWM or control white matter (Figure 4A, middle). The top network commonly associated with inactive lesions suggest that cell morphology, cellular assembly and organization, and nervous system development and function are altered (Figure 4D). These findings may highlight distinct changes that occur in affected white matter of MS brain tissue.

### 3.5 Top synergistic proteome and cellular functions altered in MS brain tissue

**FIGURE 5:**
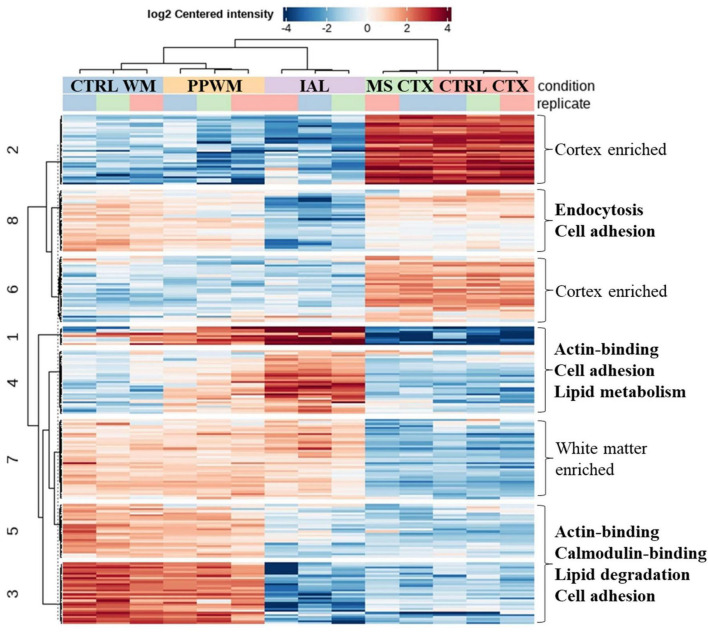
Correlated proteome changes in spatially preserved MS brain tissue. Clustered heatmap reveals coordinated changes in MS tissue. The top spatially altered proteome changes in MS brain tissue when compared to control brains. Significantly altered proteins (adjusted *p*-value < 0.05 and fold change ≥ 2) with similar patterns were grouped into 8 clusters. Clusters with similar trends (e.g., Clusters 1/4 and 3/5) were evaluated for key altered functions, which is shown on the right side of the heatmap. Cluster 7 and Clusters 2/6 were considered to be white matter enriched and cortex enriched, respectively, which had no apparent perturbations in MS tissue. CTRL, control; CTX, cortex; IAL, inactive lesion; MS, multiple sclerosis; PPWM, periplaque white matter.

To better represent proteins with similar expression pattern changes in chronic MS brain tissue, we performed cluster analysis of the top differentially expressed proteins (adjusted *p*-value < 0.05 and fold change > 2, Figure 5, see Supplementary Table 2 for protein names in each cluster). As expected, proteins were identified that were either enriched in the cortex (Clusters 2 and 6) or white matter (Cluster 7), which were largely unaffected in MS brain tissue. Clusters 3 and 5 had reduced protein expression in the inactive lesions compared to PPWM and control white matter. Key molecular functions affected were actin-binding and calmodulin-binding associated with altered biological processes including lipid degradation and cell adhesion. Interestingly, Clusters 1 and 4 have an apparent gradual increase in protein expression of affected MS white matter tissue with the greatest abundance in inactive lesions followed by PPWM and then control white matter. A single molecular function, actin-binding, was associated with Clusters 1 and 4, which may be linked to the biological processes cell adhesion and lipid metabolism. Taken together, Clusters 3/5 and 1/4 highlight dynamic changes associated with actin, cell adhesion, and lipid homeostasis in MS lesions. Lastly, Cluster 8 appears to contain proteins ubiquitously expressed in the brain, which are reduced in inactive lesions and seem largely unaffected in PPWM. While no molecular functions were significantly associated with Cluster 8, these proteins may be involved in processes including endocytosis and cell adhesion. Overall, these findings highlight distinct synergistic proteome changes in MS brain tissue, which appear to strongly affect cell adhesion and lipid homeostasis.

### 3.6 Prediction of upstream regulators affected in chronic MS inactive lesions

To gain insight into possible upstream causal factors that may be, in part, driving the altered proteome changes observed in inactive white matter lesions when compared to control white matter, we utilized three tools including KEA3 for kinase prediction (Kuleshov et al., 2021), ChEA3 for transcription factors (Keenan et al., 2019), and IPA for the activity status of biomolecules and chemicals (see materials and methods) (Krämer et al., 2013). Table 1 highlights the top 10 enriched kinases, transcription factors, and activated/inhibited molecules (five each). Several predicted upstream factors are indeed implicated in pathways that are associated with MS including myelination (AKT1, BDNF, EGFR, FYN, LAMA4, MYRF, OLIG1, OLIG2, PTK2, SMAD3, SOX2, SOX8, SRC, and TCFL2), integrin signaling and focal adhesions (ABL1, AKT1, EGFR, FYN, LAMA4, PAK1, PRKCA, PTK2, SRC, and TTN), and axon guidance (ABL1, AKTT1, BDNF, CDK5, FYN, PAK1, PRKCA, PTK2, and SRC). Of potential interest may be the epidermal growth factor receptor (EGFR) as it was identified amongst the top causal factors predicted for KEA3 and IPA (Table 1). More so, one of the top ten transcription factors, ARNT2, has been shown previously to upregulate the gene expression of EGFR (Liu et al., 2003). Overall, these inferred upstream regulators may provide insight into biological mechanisms regulating the altered protein expression patterns observed in MS lesions.

**TABLE 1:** Upstream regulators.

Method^a^	Kinases	Mean rank score^b^	Protein overlaps^c^
KEA3	PRKCA	10.55	222
	EGFR	18.36	299
	FYN	19.64	221
	PAK1	21.36	123
	SRC	28.09	310
	PTK2	30.36	155
	CDK5	33.82	183
	AKT1	34.91	327
	ABL1	42.45	170
	TTN	45.25	112
**Method^a^**	**Transcription factors**	**Mean rank score^b^**	**Protein overlaps^c^**
ChEA3	NKX62	3	87
	MYRF	5	87
	SOX8	9.333	101
	OLIG1	11.67	96
	NACC2	22.5	69
	OLIG2	25.5	140
	BHLHE41	35	68
	CSRNP3	42.5	48
	ST18	44	86
	ARNT2	44	85
**Method^a^**	**Inhibited regulator**	**Activation z-score^b^**	**Protein overlaps^c^**
IPA	TCF7L2	−4.93	47
	SOX2	−4.121	36
	estrogen receptor	−3.302	14
	BDNF	−3.289	29
	LAMA4	−3.162	10
	**Activated regulator**	**Activation z-score^b^**	**Protein overlaps^c^**
	cuprizone	4.076	29
	SMAD3	3.074	16
	hexachlorobenzene	2.813	8
	EGFR	2.776	33
	thioacetamide	2.728	23

^a^Method used to detect upstream regulators (KEA3, Kinase Enrichment Analysis 3; ChEA3, ChIP-X Enrichment Analysis 3; IPA, Ingenuity Pathway Analysis)

^b^Mean rank score = Composite score calculated across all libraries used. Lower values are more significant. Activation z-score = Predicted activation (positive value) or inhibition (negative value) score based on protein expression data.

^c^Overlap of proteins in dataset with upstream regulator.

## 4 Discussion

In our study, we identified spatial alterations in the white matter proteins of chronic MS individuals when compared to control brain tissue. Proteomics analysis of inactive lesions and PPWM associated differentially expressed proteins with myelination, oligodendrocyte development, metabolism, cell adhesion, and cellular structure. Proteomics can provide valuable insight into disease mechanisms; however, studies assessing spatial changes in the proteome of MS brain tissue remains sparse. Our studies not only demonstrate the feasibility of utilizing archived brain tissue for proteomics analysis but may have relevance to underlying pathophysiological mechanisms that prevent recovery in the MS brain.

### 4.1 Focal adhesions and ECM-receptor interaction

The top altered pathways identified in MS chronic inactive white matter lesions compared to control white matter included focal adhesion, ECM-receptor interaction, and metabolic pathways. The focal adhesion pathway focuses on cell-matrix contact points critical for cell signaling involved in the regulation of cell shape, motility, gene expression, proliferation, differentiation, and survival (Wehrle-Haller, 2012). Similarly, the ECM-receptor interaction pathway utilizes transmembrane molecules to modulate tissue structure and function and cellular activities including adhesion (Kerrisk et al., 2014). While the focal adhesion and ECM-receptor interaction pathways have distinct functions, many of the proteins involved overlap resulting in an intricate relationship (Wehrle-Haller, 2012; Kerrisk et al., 2014). Adhesion and ECM factors are crucial for oligodendrocyte function (Baron et al., 2005). Cytoskeletal-ECM alterations have long been implicated in the failure to remyelinate in chronic inactive MS lesions (Elovaara et al., 2000; Van Horssen et al., 2007). Fibronectin aggregation in MS lesions was associated with a failure to remyelinate (Stoffels et al., 2013). In a toxin-induced demyelinating rat model, fibronectin was found to be increased within lesions, which declined with remyelination. In chronic MS lesions (active and inactive), fibronectin levels were elevated, which were not seen in control tissue. Fibronectin aggregates were not observed in a rat model of toxin-induced demyelination but was seen in a chronic relapsing experimental autoimmune encephalomyelitis (EAE) model during the relapse phase suggesting that aggregation was due to inflammation (Stoffels et al., 2013). These observations highlight the importance of adhesion-ECM components in the pathogenesis of MS.

In our studies, the pathways focal adhesion and ECM-receptor interaction consisted of 27 and 18 differentially expressed proteins, respectively, in MS chronic inactive lesions when compared to control white matter. Of these proteins, a total of 30 unique proteins were amongst the two pathways. Six proteins were members of the collagen superfamily (collagens I, IV, and VI) of which were all upregulated in the MS chronic inactive lesions. Intriguingly, collagen VI types 1, 2, and 3 were amongst the top four (out of 30) adhesion-ECM proteins altered. Collagen VI is an ECM protein that forms heterotrimeric filaments impacting cell structure and intracellular signaling. In a previous study, the gene expression of collagen I, III, and V were found to be increased in MS active and inactive white matter lesions when compared to normal controls, which may be crucial for proper immune functions (Mohan et al., 2010). Interestingly, collagen may inhibit migration of oligodendrocyte precursor cells (Milner et al., 1996), which may in turn impact oligodendrocyte function and myelination. Integrins and their functions as cell adhesion molecules can facilitate immune cell trafficking in the brain and are recognized as key factors in the pathogenesis of MS (Ransohoff, 2007). Herein, we identified three integrin proteins significantly increased in MS chronic inactive lesions (ITGA6, ITGA7, and ITGB4). In an EAE mouse model for MS, increased expression of integrin (α6β4) was associated with increased protection against neuroinflammatory conditions (Welser et al., 2017). Furthermore, we detected increased protein expression in MS chronic inactive lesions of known ligands for integrins including collagens and laminins. Previously, integrin alpha-6 and -7 have been reported to bind laminins (Haas et al., 2017). Laminin has been shown to promote oligodendrocyte precursor cell migration, which is likely to be important for the distribution and development of oligodendrocytes (Milner et al., 1996). In another study, increased laminin gene expression was detected in demyelinated inactive lesions as compared to control brain tissue (Mohan et al., 2010), which is consistent with our findings. Taken together, our findings and others demonstrate a crucial role for cell adhesions and the ECM in the pathogenesis of MS.

### 4.2 Metabolic pathways

In our study, proteins associated with metabolic pathways were heavily perturbed in chronic inactive MS lesions compared to control white matter. Pathway analysis identified 77 proteins associated with metabolic functions in chronic inactive MS lesions. Metabolic processes in general are necessary for the generation of energy and detoxification reactions. Indeed, dyshomeostasis of metabolic processes are linked to increased oxidative stress, mitochondrial dysfunction, inflammation, and perturbed oxidative phosphorylation and glycolytic processes in MS (Mattson and Arumugam, 2018; Peruzzotti-Jametti and Pluchino, 2018; Park and Choi, 2020). Interestingly, of the 77 metabolic proteins altered in MS inactive white matter lesions, about 30% of the proteins are associated with lipid metabolism. Of these, the topmost altered protein was PIP4K2A, which is largely associated with myo-inositol metabolism. Five additional proteins involved in myo-inositol metabolism were also detected including EPHX2, MTMR2, PLCB1, PLCD1, and SYNJ2. Intriguingly, magnetic resonance spectroscopic imaging of NAWM in MS individuals detected elevated levels of myo-inositol when compared to controls, which may be due to inflammation and axonal damage (Heckova et al., 2022). In another study utilizing blood samples from patients with MS, it was suggested that inositol may be abnormally metabolized contributing towards demyelination (Holm, 1978). Taken together, our findings and others highlight the complexities of altered metabolic pathways in the pathogenesis of MS.

### 4.3 Causal associations

Hallmarks of MS include inflammation, demyelination, axon dysfunction, and metabolic alterations. Analysis of the differentially expressed proteins identified here has provided valuable insight into biological functions and pathways that may be contributing to the pathogenesis in MS brain tissue. Equally important, however, is the identification of potential upstream regulators that may be driving the proteome changes observed. These causal inferences may shed light onto biological mechanisms that influence inflammation, myelination, axon function, and metabolism. Herein we utilized three different tools to infer upstream kinases, transcription factors, chemicals, and their potential activity status. Indeed, many of the factors are known to affect processes that are perturbed in MS. For instance, many of the upstream factors are implicated in immune functions (ABL1, AKT1, FYN, SMAD3, SRC, SOX2, PAK1, and PTK2), myelination (AKT1, BDNF, EGFR, FYN, LAMA4, MYRF, OLIG1, OLIG2, PTK2, SMAD3, SOX2, SOX8, SRC, and TCFL2), axon guidance (ABL1, AKTT1, BDNF, CDK5, FYN, PAK1, PRKCA, PTK2, and SRC), and metabolism (AKT1, EGFR, SRC, and TCF7L2). Of particular interest may be EGFR as it was identified by KEA3 and IPA. The EGFR pathway facilitates extracellular signals to modulate intracellular processes that affect proliferation, protein synthesis, cytokines, cell survival, endocytosis, and development. In MS, EGFR signaling is linked to remyelination failure, nervous system development, and modification of the ECM (Rivera et al., 2022), which are among the top pathways altered in inactive lesions compared to controls identified in our study. Thus, utilizing causal inference for the identification of upstream factors may provide valuable insight into biological factors impacting the pathophysiological mechanisms driving MS.

### 4.4 Top protein alterations in MS inactive lesions

In our study, the top proteins altered in inactive lesions compared to control white matter included PPP1R14A, ERMN, SIRT2, CARNS1, and MBLAC2 (ranked in order by *p*-value). All five proteins were significantly reduced in inactive lesions.

The protein PPP1R14A (Protein phosphatase 1 regulatory subunit 14A) was identified as an inhibitor of smooth muscle myosin phosphatase (Yamawaki et al., 2001). It has been detected in the brain where it may function in long-term synaptic depression, cytoskeletal assembly, vesicular trafficking, and endocytosis and/or exocytosis (Zemlickova et al., 2004). However, any direct link to MS is limited.

The protein ERMN (Ermin) is an actin-binding protein involved in the maintenance of the myelin sheath. A rare inactivating mutation in ERMN was discovered in patients presenting with MS (Ziaei et al., 2022). Furthermore, ERMN has been associated with remyelination of MS lesions (Ahmad et al., 2021).

Sirtuins are key players in inflammation, metabolism, oxidative stress, myelination, and apoptosis amongst other biological processes. The sirtuin family, including SIRT2 (NAD-dependent protein deacetylase sirtuin-2) has been implicated in neurodegenerative processes (Lu et al., 2023), which may have relevance in MS (Foolad et al., 2019).

The protein CARNS1 (Carnosine synthase 1) catalyzes the synthesis of carnosine, which has antioxidant and antiinflammatory properties (Chasovnikova et al., 1990; Bellia et al., 2011). In a study by Spaas and colleagues (Spaas et al., 2023), they show in human MS brain tissue that CARNS1 immunoreactivity colocalized with OLIG2^+^ in NAWM. In the MS lesion CARNS1 was greatly diminished (Spaas et al., 2023), which is in agreement with our data. Spaas and colleagues further showed that Carns1 deficiency exacerbated neuroinflammation and acrolein (a lipid-derived reactive carbonyl) in the experimental autoimmune encephalomyelitis (EAE) MS mouse model (Spaas et al., 2023). This supports a beneficial role of CARNS1 in MS.

Acyl-coenzyme A thioesterase (MBLAC2) has been shown to have robust thioesterase activity toward palmitoyl-CoA resulting in the formation of palmitate and CoA (Malgapo et al., 2021). Interestingly, inhibition of MBLAC2 results in the accumulation of extracellular vesicles (Lechner et al., 2022). Evidence suggests that extracellular vesicles are important factors in MS affecting inflammatory processes and lesion repair (Sáenz-Cuesta et al., 2014). These findings may highlight MBLAC2 as a key factor in MS.

Taken together, our data and others provide evidence for key factors in the pathophysiology of MS. While future studies will be needed to delineate their exact roles in MS, these findings may further help with identifying new pathways and networks that can be targeted by therapeutics.

### 4.5 Comparison to other proteomics studies in MS brain tissue

To date, proteomics analysis of MS brain tissue is limited (Elkjaer et al., 2022). In a study by Newcombe et al. (2005), they interrogated proteome changes in the brain tissue of three MS patients and two controls. Control white matter, MS plaques, and plaque-adjacent white matter (i.e., PPWM) was used. Principal component analysis of the 109 proteins shows variation between MS and control tissue, however, no details on altered proteins were given.

Han et al. compared acute plaques (containing myelin fragments), chronic active plaques, chronic plaques (few or no inflammatory cells), and controls (two samples per group) (Han et al., 2008). The authors identified 2,302 proteins in MS tissue and 1,492 in controls. They found 158, 416, and 236 proteins that were unique to acute, chronic active, and chronic plaques, respectively. From the 236 unique proteins identified by Han and colleagues, five overlapped in our study with proteins significantly altered in MS inactive lesions compared to control white matter (ACY1, CYB5R2, HPCA, PTGR2, AND SNPH). Of the five identified in our study, HPCA was the most significantly altered in inactive plaques (decreased about 5-fold). The protein HPCA (Neuron-specific calcium-binding protein hippocalcin) is a neuronal calcium sensor, which may promote gliogenesis by signaling through STAT3 (Kang et al., 2016). In our study, STAT3 was significantly increased in inactive lesions compared to controls. These observations may have significance with proper neurogenesis and remyelination (Kang et al., 2016; Steelman et al., 2016).

Another study by Fissolo et al. identified peptides bound to major histocompatibility complexes I and II in brain autopsy tissue (no regional information provided) from eight patients with MS (Fissolo et al., 2009). The authors identified 174 source proteins of which overlapped with nine proteins significantly altered in inactive lesions when compared to PPWM within our study including ABCA2, BIN1, FA2H, LGALS3BP, MBP, PLIN3, SIRT2, STMN1, and TUBA1B. Of these, SIRT2 was the most significantly decreased in inactive lesions (about 18-fold). The protein SIRT2 is a member of the sirtuin family, which may have a role in MS pathology (Foolad et al., 2019). Taken together, these observations may highlight proteins more readily targeted by autoimmune processes.

A study by Ly et al. and colleagues examined MS chronic active lesions and periplaque white matter from three patients identifying 428 unique proteins (Ly et al., 2011). The authors highlighted eight proteins of interest altered in MS brain tissue including GFAP, CNTN1, HAPLN2, MAG, BCAN, ENO1, and PRDX6. In our study, GFAP, CNTN2, HAPLN2, MAG, BCAN, and PRDX6 had significant alterations in inactive lesions when compared to control white matter. Three of these proteins (HAPLN2, MAG, and PRDX6) were also found to be significantly altered between inactive lesions and PPWM in our study. Of these, our results indicate that HAPLN2 had the most significant alteration in inactive lesions compared to control white matter (29-fold decrease in lesion). A 15-fold decrease of HAPLN2 was detected in inactive lesions when compared to PPWM. Ly and colleagues noted a 49-fold decrease of HAPLN2 in chronic active lesions compared to normal white matter (Ly et al., 2011). The protein HAPLN2 (Hyaluronan and proteoglycan link protein 2) is important in the organization of the extracellular matrix in the brain and is implicated in neurodegenerative disease (Wang et al., 2019). In mice, expression of Hapln2 was observed in myelinated fiber tracts, which may be important for neuronal conduction (Oohashi et al., 2002). Taken together, disruption of the extracellular matrix and HAPLN2 likely plays an important dynamic role in neural protection against MS and other neurodegenerative diseases.

Our study and others have highlighted the utility of proteomics analysis in human MS brain tissue for the potential identification of key pathological factors. Unfortunately, there is no consensus in proteomics strategies to study MS brain tissue leading to variations in approach and mapping of histological features for analysis. Nonetheless, our findings and others provide valuable insight into underlying mechanism of MS.

### 4.6 Periplaque white matter alterations

A study by Nataf et al. examined the gene expression level of PPWM, which demonstrated that myelin genes and TGF-beta related factors were affected regardless of the lesion activity (Nataf et al., 2022). The authors report that myelin-related genes (MBP, MOBP, PLP1, MOG, MAG, NDRG1, OLIG1, and OLIG2) were down in the rim of active lesions compared to control white matter. They moved on to note that myelin-related genes were less affected in PPWM, but MAG, NDRG1, OLIG1, and OLIG2 gene expression were still decreased (fold-change > 1.2) and remained significantly affected compared to control white matter. In our study, we noted nine myelin-related proteins that were significantly decreased (fold-change > 2) in inactive lesions compared to control white matter (CLDN11, CNP, ERMN, MAG, MBP, MOBP, MOG, PLP1, SIRT2) that which were partially recovered or unaffected in MS PPWM. Nataf and colleagues further identified significantly increased genes related to the “signaling by TGF-beta receptor complex” in PPWM irrespective of plaque activity (Nataf et al., 2022). They noted 24 genes significantly upregulated in “silent periplaque”. While we detected several overlapping proteins in our data (SMAD2, PARP1, UBE2D3, UCHL5, PPP1CB/C, PPM1A, XPO1, and NCOR1), we did not detect significant changes at the protein level in PPWM compared to inactive lesions. While future studies will require precise comparisons, these findings show some overlap between gene expression and protein level profiles in MS with the greatest correlation between myelin-related genes.

Graumann and colleagues carried out a microarray analysis of MS NAWM and control white matter from post-mortem brain tissue (Graumann et al., 2003). The authors identified approximately 175 significantly differentially expressed genes in MS NAWM compared to control white matter. Of those, 79 genes were detected at the protein level in our study. Unfortunately, the MS tissue utilized in our study did not contain NAWM. However, compared to control white matter, significant changes in the proteins PDE2A, GSTM1, and PCSK2 in our study are consistent with altered gene expression as detected by Graumann et al. (2003).

Phosphodiesterase 2A is capable of hydrolyzing and breaking down cAMP and cGMP. Allosteric binding of cGMP to PDE2A can increase the breakdown of cAMP, thus, creating a crosstalk between the two molecules. Furthermore, cAMP is a well-known regulator of immune functions (Raker et al., 2016). In a study by Kurelic et al., they show that upregulation of PDE2A can alter T cell activation by changing cGMP/cAMP ratios (Kurelic et al., 2021). Moreso, in the EAE mouse model, they detected a 2-fold increase in gene expression of Pde2a at the peak of disease severity. This suggested that Pde2a played a role in T cell activation after autoantigen immunization in the EAE mouse model (Kurelic et al., 2021). Taken together, our data and others support a role for PDE2A in MS possibly affecting autoimmune functions in PPWM.

Glutathione S-transferase Mu 1 (GSTM1) is an enzyme involved in detoxification against toxic (Bhattacharjee et al., 2013). Oxidative damage and the release of toxic products are known to be involved in the pathogenesis of MS lesions (Popescu et al., 2013). While no clear genetic association of GSTM1 is established with MS, it may have a implications on detoxification in a gender-specific manner (Stavropoulou et al., 2007). In our study, we see a significant increase of GSTM1 protein in both inactive lesions and PPWM relative to controls. This would suggest an increased presence of toxic products in MS brain tissue supporting an important role for GSTM1 detoxification processes.

The protein neuroendocrine convertase 2 (PCSK2) is a member of the subtilisin-like proprotein convertase family, which are known for the maturation of prohormones, neuropeptides, and other precursor-derived proteins (Rouillé et al., 1995). A study by Shiryaev and colleagues suggests PCSK2 is involved in an inflammatory proteolytic pathway in which matrix metalloproteinases (MMP-25 in particular) are activated causing demyelination in MS (Shiryaev et al., 2009). The authors demonstrated an increase in PCSK2 gene and protein expression in LPS-activated macrophages. It was proposed that PCSK2 can cleave and activate MMP-25, which can result in the breakdown of myelin similar to what is seen in autoimmune and inflammatory processes in MS (Shiryaev et al., 2009). Given the elevated protein expression of PCSK2 detected in PPWM of our study and observations made by others, it is plausible that inflammatory processes activate proteolytic pathways and may play a role in autoimmune functions making these promising targets in MS.

In our study, we detected a subset of 65 proteins that reached significant changes in PPWM compared inactive lesions that did not reach significant changes when comparing control white matter to inactive lesions. Of these 65 proteins, the top three altered proteins in PPWM compared to inactive lesions consisted of ABCA8 (ATP-binding cassette sub-family A member 8), PRKACA (cAMP-dependent protein kinase catalytic subunit alpha), and PDE2A (cGMP-dependent 3,5-cyclic phosphodiesterase; discussed above). All three proteins had increased expression in the PPWM (fold-change > 2, *p*-value < 0.01), and are associated with metabolic activities.

The protein ABCA8 is a lipid transporter that can modulate the levels of high-density lipoprotein cholesterol (Liu et al., 2022). Furthermore, ABCA8 has been shown to stimulate sphingomyelin production in oligodendrocytes supporting a role in lipid metabolism and myelin maintenance (Kim Woojin et al., 2013). Moreso, regulation of the ABCA8 gene during oligodendrocyte differentiation was tightly linked to MS susceptibility loci (Dugas et al., 2006). Our study and others provide evidence for an intrinsic role of ABCA8 in MS.

As mentioned in section “Focal adhesions and ECM-receptor interaction”, fibronectin aggregation is linked to incomplete remyelination in MS lesions (Stoffels et al., 2013). Additional studies by Qin et al. show in a cuprizone mouse model that treatment with the ganglioside GD1a stimulates oligodendrocyte precursor cell proliferation and differentiation by overcoming the inhibitory effects of fibronectin aggregates (Qin et al., 2017). Their findings indicated GD1a stimulation was due to the activation of protein kinase A. As we detected a significant increase in the protein PRKACA in PPWM compared to inactive lesions, but not in control white matter compared to inactive lesions, our results and others support a role for protein kinase A in the maintenance of myelin in affected MS white matter.

Identification of early changes in MS continues to be an area of high interest. However, little is known about early molecular mechanisms that lead to the formation of lesions in MS. Nonetheless, continued efforts to correlate changes in imaging, protein, and gene expression profiles, among others, will aid greatly in the efforts to identify early molecular mechanisms in MS.

## 5 Conclusion

In MS, one key failure is the inability to completely remyelinate after injury. This is likely due to the imbalance between processes that are destructive and restorative. As shown here, profound proteome changes occur within chronic inactive white matter lesions and the PPWM of MS individuals. Spatial alterations further suggests that distinct proteome changes occur between inactive lesions and PPWM. While our study was limited to a small number of cases, proteome changes detected herein complement previous observations yet provide valuable insight into possible novel protein candidates affecting myelinogenesis and other processes in MS. As women are more likely to develop MS than men, it is important to consider how sex-specific changes can influence outcomes detected in various studies (Voskuhl, 2020). Future studies utilizing larger cohorts will help validate changes identified here while further allowing for the detection of possible patient-specific changes, which may be affected by sex, age, medication, disease status, etc. The strategies presented here will help aid in generating proteomics data from FFPE tissue for the continued investigation of pathophysiological mechanisms in MS and development of targeted therapeutics.

## Data availability statement

The data presented in the study are deposited in the ProteomeXchange Consortium via the PRIDE partner repository, accession number PXD047800.

## Ethics statement

The studies involving humans were approved by the Mayo Clinic Internal Review Board. The studies were conducted in accordance with the local legislation and institutional requirements. The participants provided their written informed consent to participate in this study.

## Author contributions

JW: Conceptualization, Data curation, Formal analysis, Investigation, Methodology, Visualization, Writing−original draft, Writing−review and editing. KM: Data curation, Methodology, Writing−review and editing. BN: Formal analysis, Methodology, Writing−review and editing. WS: Formal Analysis, Methodology, Writing−review and editing. YG: Data curation, Writing−review and editing. AK-L: Data curation, Writing−review and editing. AP: Conceptualization, Data curation, Methodology, Writing−review and editing. CL: Conceptualization, Data curation, Formal Analysis, Funding acquisition, Methodology, Visualization, Writing−review and editing.

## Footnotes

1
https://digitalinsights.qiagen.com/IPA
