# Supplementary material for: Proteomics analysis of periplaque and chronic inactive multiple sclerosis lesions
Source: Front Mol Neurosci. 2024 Aug 21;17:1448215. doi: 10.3389/fnmol.2024.1448215 (PMC11371774; doi:10.3389/fnmol.2024.1448215)
Supplement: Supplementary file 1 [file Data_Sheet_1.docx]

Supplementary Material

**Proteomics analysis of chronic inactive multiple sclerosis lesions**

**Jordan M. Wilkins, Kiran K. Mangalaparthi, Brian C. Netzel, William A. Sherman, Yong Guo, Alicja Kalinowska-Lyszczarz, Akhilesh Pandey, Claudia F. Lucchinetti^*^**

*** Correspondence:** Claudia F. Lucchinetti, M.D: [claudia.lucchinetti@austin.utexas.edu](mailto:claudia.lucchinetti@austin.utexas.edu)

**Supplementary table 1**

**Supplemental table - Clinical summary**

| **Group** | **Age^a^** | **Sex** | **Disease course^b^** | **Disease duration^c^** |
| --- | --- | --- | --- | --- |
| Control | 50 | M | NA | NA |
|  | 68 | M | NA | NA |
|  | 20 | M | NA | NA |
|  |  |  |  |  |
| MS | 57 | F | SPMS | 22 |
|  | 60 | M | RRMS | 11 |
|  | 68 | F | SPMS | 24 |

^a^ Age at death (in years).

^b^ Disease course at death.

^c^ Disease duration at death (in years).

Abbreviations: F, female; M, male; MS, multiple sclerosis; NA, not applicable.

**Supplementary table 2**

**Supplemental table - Altered proteins in each cluster from Figure 5**

| **Cluster 1** | **Cluster 2** | **Cluster 3** | **Cluster 4** | **Cluster 5** | **Cluster 6** | **Cluster 7** | **Cluster 8** |
| --- | --- | --- | --- | --- | --- | --- | --- |
| SYNM | ICAM5 | PLEKHB1 | S100A7A | NELL2 | TTC9 | RANBP6 | SYNJ2BP |
| COL6A1 | ATCAY | HDAC11 | MECR | HTATIP2 | XP32 | SERPINA1 | PITRM1 |
| COL6A3 | GRIA2 | MYO1D | NEK9 | AKTIP | KCNIP4 | GTPBP3 | TFRC |
| COL1A2 | SYNPO | GLTP | GMPR | ABHD17B | FBXL16 | IQGAP1 | NAPEPLD |
| FLNC | SV2A | LIPE | ARHGEF6 | ICOSLG | SCAMP1 | H2BFS | SLC12A2 |
| GFAP | ARFGAP1 | PLIN3 | LYPLA1 | SEPTIN4 | MADD | EPB41L2 | RHOB |
| CFAP43 | GRIN1 | HAPLN2 | COL21A1 | PLCL1 | ATP2A2 | CPPED1 | BIN1 |
| TNC | HOMER1 | MAG | COL6A2 | GNG7 | PRKAR2B | LMNA | GNAI1 |
| AQP1 | RAB3A | ENPP6 | GYG1 | FAM107B | PPFIA3 | QKI | TUBB8 |
|  | ATP2B3 | WDR59 | ADH5 | QDPR | CADM1 | LLGL1 | FSCN1 |
|  | ACTN2 | CNP | PLEC | ELMO1 | GGT7 | CRYAB | PRNP |
|  | SGIP1 | SLC44A1 | ABHD14B | LSS | KCTD16 | HIST1H1B | NFASC |
|  | DLG4 | LGI3 | VIM | LGALS3BP | DLGAP3 | CLIC4 | RTN4 |
|  | SYN3 | MOBP | PLCD1 | VCAN | GABRB2 | GSN | STMN1 |
|  | PDE2A | N4BP2L1 | PDE12 | SEPTIN8 | AVL9 | HSPA2 | TARSL2 |
|  | SLC1A2 | ERMN | VCL | ENDOD1 | GABBR2 | HIST1H2BO | SRCIN1 |
|  | ATP6AP1 | SIRT2 | FLNA | FMNL2 | SLC6A17 | HNRNPF | TPD52 |
|  | SLC30A3 | PPP1R14A | CAPNS1 | CD9 | SYT7 | RP2 | SVIP |
|  | PLXNA4 | ANLN | IGKV3OR2-268 | RHOG | GABBR1 | CSRP1 | GAPVD1 |
|  | DMXL2 | PTMA | IGHG1 | CFL2 | FAHD1 | TJP2 | PTPRS |
|  | ATP6V0D1 | PIP4K2A | IGKV A18 | MARCKSL1 | KIAA1217 | RDX | SBF1 |
|  | SYNGR1 | INF2 | IGLC6 | GLIPR2 | CCK | TPPP3 | AK5 |
|  | ATP2B1 | CARNS1 | IGJ | JAM3 | ADRBK1 | FGF1 | ADAP1 |
|  | PSD3 | BCAS1 | IGHA1 | RAP1A | NRXN1 | GNA13 | TMEM163 |
|  | VAMP2 | OPALIN | IGKV3D-11 | FAM63A | CELF2 | DDAH2 | SNCB |
|  | ATP6V0A1 | CLDN11 | SHMT1 | PLA2G16 | EARS2 | MPST | IGSF8 |
|  | SYN2 | PLLP | GSTM3 |  | TSFM | CBR3 | MBLAC2 |
|  | SYNGR3 | P2RX7 | LGALS3 |  | CSNK1D | FLNB | TPPP |
|  | SV2B | OMG | GSTM1 |  | RALGAPA1 | TAX1BP3 | HPCA |
|  | SYP | MAP6D1 | SULT1A1 |  | FXYD1 | ALAD | NCAN |
|  | TTC7B |  | TECPR1 |  | CCDC177 | RNH1 |  |
|  | RAB3C |  |  |  | SCG3 | SBDS |  |
|  | SLC17A7 |  |  |  |  | SEPTIN10 |  |
|  | KIAA0513 |  |  |  |  | CA2 |  |
|  |  |  |  |  |  | MYH14 |  |
|  |  |  |  |  |  | P2RY12 |  |
|  |  |  |  |  |  | TSC22D4 |  |
|  |  |  |  |  |  | AGT |  |
|  |  |  |  |  |  | L3HYPDH |  |

**Supplementary figure 1**

**Inactive lesion vs Control (white matter)**

**
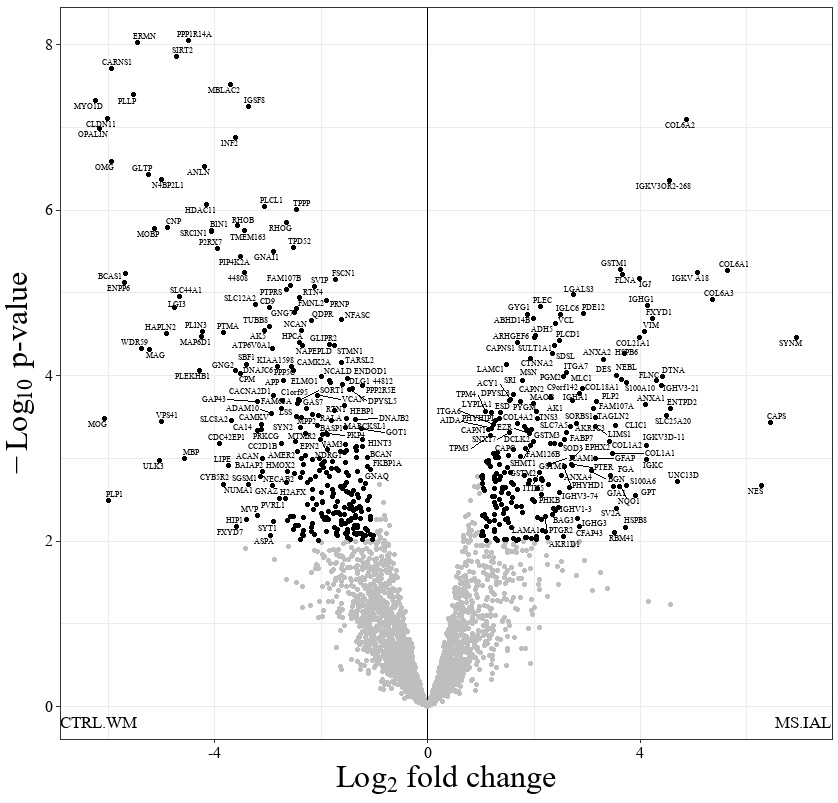
**

**Supplementary figure 1.** Proteome changes in MS inactive white matter lesions compared to control white matter. A volcano plot highlighting significantly altered proteins in MS inactive lesions when compared to control white matter tissue (p-value < 0.01 and fold-change ≥ 2).

**Supplementary figure 2**

**IAL vs CWM**

**
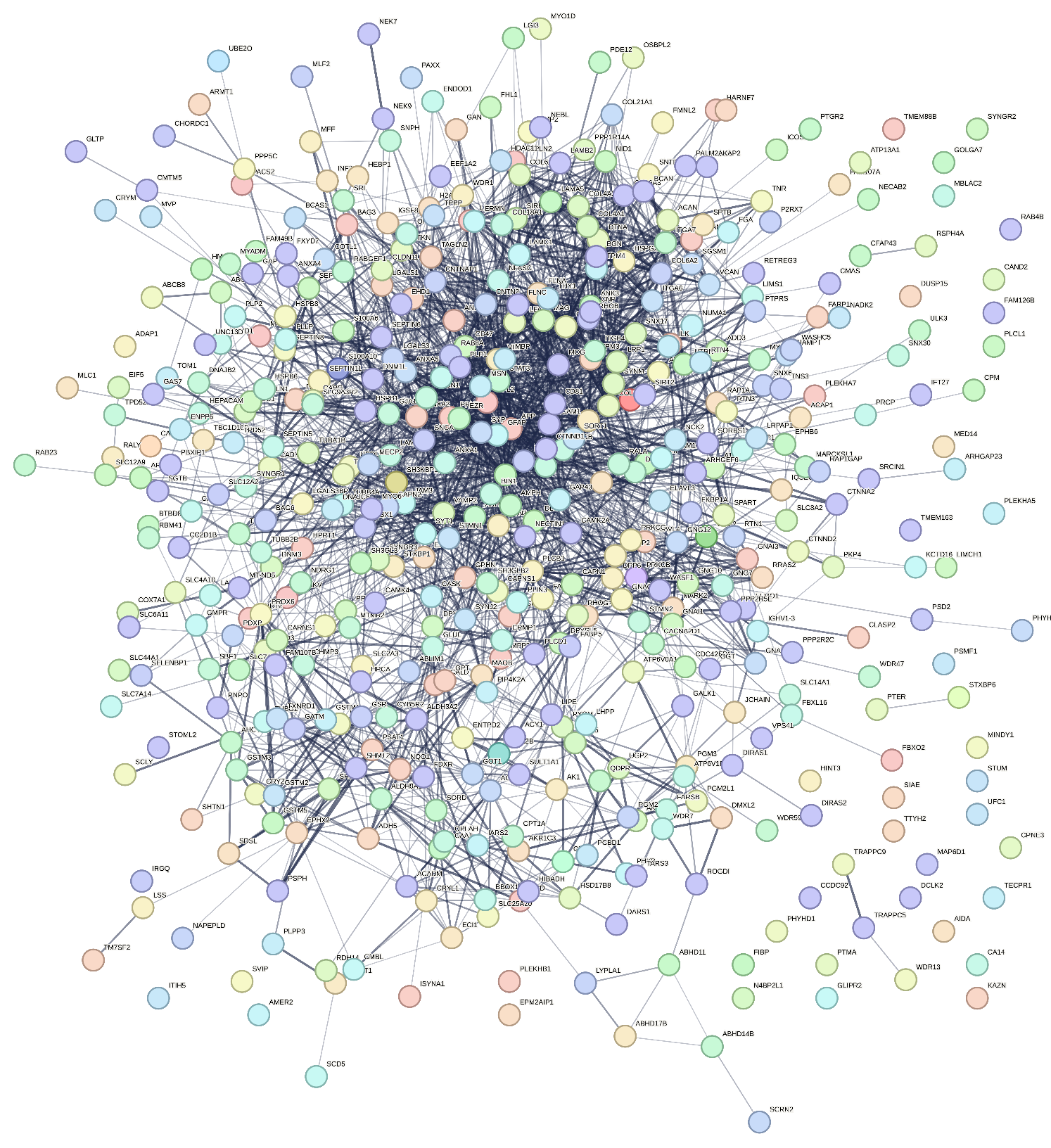
**

**Supplementary figure 2.** Protein-protein interaction network of all differentially expressed proteins in MS inactive white matter lesions compared to control white matter (p-value < 0.01 and fold-change ≥ 2).

**Supplementary figure 3**


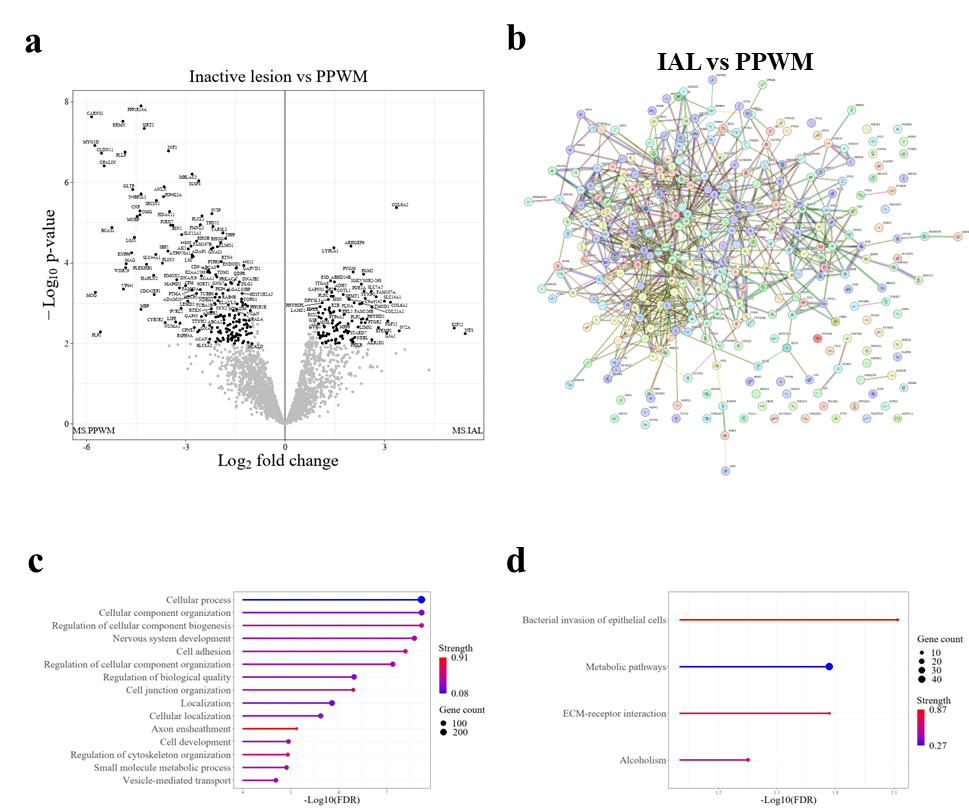


**Supplementary figure 3.** Proteome alterations in MS periplaque white matter. Significantly altered proteins in PPWM when compared to inactive lesions. Differentially expressed proteins had a p-value < 0.01 and fold-change ≥ 2. **a** A volcano plot of significantly altered proteins in PPWM compared to inactive lesions. **b** Protein-protein interaction network of differentially expressed proteins. **c** Enrichment of biological functions. **d** Enrichment of pathways. Gene count and Strength were determined by the STRING application in **c** and **d**. Abbreviations: PPWM, periplaque white matter; IAL, inactive lesion; MS, multiple sclerosis.
